# PlantConnectome: A knowledge graph database encompassing >71,000 plant articles

**DOI:** 10.1093/plcell/koaf169

**Published:** 2025-07-23

**Authors:** Shan Chun Lim, Manoj Itharajula, Mads Harder Møller, Rohan Shawn Sunil, Kevin Fo, Yu Song Chuah, Herman Foo, Emilia Emmanuelle Davey, Melissa Fullwood, Guillaume Thibault, Marek Mutwil

**Affiliations:** School of Biological Sciences, Nanyang Technological University, 60 Nanyang Drive, Singapore 637551, Singapore; School of Biological Sciences, Nanyang Technological University, 60 Nanyang Drive, Singapore 637551, Singapore; Section of Plant Biochemistry, Department of Plant and Environmental Sciences, University of Copenhagen, Thorvaldsensvej 40, Frederiksberg, Copenhagen 1871, Denmark; School of Biological Sciences, Nanyang Technological University, 60 Nanyang Drive, Singapore 637551, Singapore; School of Biological Sciences, Nanyang Technological University, 60 Nanyang Drive, Singapore 637551, Singapore; School of Biological Sciences, Nanyang Technological University, 60 Nanyang Drive, Singapore 637551, Singapore; School of Biological Sciences, Nanyang Technological University, 60 Nanyang Drive, Singapore 637551, Singapore; School of Biological Sciences, Nanyang Technological University, 60 Nanyang Drive, Singapore 637551, Singapore; School of Biological Sciences, Nanyang Technological University, 60 Nanyang Drive, Singapore 637551, Singapore; School of Biological Sciences, Nanyang Technological University, 60 Nanyang Drive, Singapore 637551, Singapore; School of Biological Sciences, Nanyang Technological University, 60 Nanyang Drive, Singapore 637551, Singapore; Section of Plant Biochemistry, Department of Plant and Environmental Sciences, University of Copenhagen, Thorvaldsensvej 40, Frederiksberg, Copenhagen 1871, Denmark

## Abstract

One of the main quests in plant biology is understanding how gene products and metabolites work together to form complex networks that drive plant development and responses to environmental stimuli. However, the ever-growing volume and diversity of scientific literature make it increasingly challenging to stay current with the latest advances in functional genetics studies. Here, we tackled this challenge by deploying the text-mining capacities of large language models to process over 71,000 plant biology abstracts. Our approach presents nearly 5 million functional relationships between 2.4 million biological entities—genes or gene products, metabolites, tissues, and others—with a high accuracy of over 85%. We encapsulated these findings in the user-friendly database PlantConnectome and demonstrated its diverse utility by providing insights into gene regulatory networks, protein–protein interactions, and stress responses. We believe this innovative use of artificial intelligence (AI) in the life sciences will allow plant scientists to keep up to date with the rapidly growing corpus of scientific literature. PlantConnectome is available at https://plant.connectome.tools/.

## Teaser

PlantConnectome uses large language models to extract ∼5 million functional relationships between genes, metabolites, subcellular compartments, treatments, and other entities.

## Introduction

Despite decades of research, only ∼15% of thale cress (*Arabidopsis thaliana)*'s genes have been comprehensively characterized (captured by Gene Ontology terms with experimental evidence), and the rate of new articles reporting gene functions in Arabidopsis has dropped to <30% in 2023 (∼380 GO terms assigned) since the peak in 2008 (∼1,200 GO terms assigned; Sunil et al. 2024). Due to the time-consuming experiments and increased requirements to publish in premier journals, the time needed to characterize a gene can take several years. Thus, choosing which gene to start characterizing requires a strong hypothesis, which can often be based on previous literature, even in unrelated fields. However, staying up to date with the continuously growing scientific literature and integrating the numerous pieces of the gene function puzzle can be time-consuming and limit our ability to form strong hypotheses.

Alternatively, computational gene function prediction can suggest which genes have a specific function and are invaluable in generating new gene function hypotheses (Brown et al. 2005; Persson et al. 2005). Predicting gene function requires 2 components: (i) omics data that captures gene properties (e.g. coding sequence, expression patterns, and protein structure) and (ii), gold standard data (i.e. genes with experimentally verified functions) (Radivojac et al. 2013; Rhee and Mutwil 2014). The omics data is firstly used to connect uncharacterized and characterized genes based on sequence or expression similarity. Then, uncharacterized genes are labeled according to the functions of the characterized genes (i.e. the gold standard data) to which they were connected (Rhee and Mutwil 2014).

Nonetheless, gene function prediction remains highly challenging due to the complexity and vastness of biological data, plateauing our understanding of gene functions (Radivojac et al. 2013). Specifically, establishing the gold standard necessitates manual, work-intensive extraction of gene functional information from scientific articles (Oughtred et al. 2021), preventing public repositories that harbor the gold standard data, such as BioGRID (protein–protein interactions, or PPIs) and AGRIS (gene regulatory networks, or GRNs) (Yilmaz et al. 2011; Oughtred et al. 2021), from keeping up to date with state-of-the-art knowledge. Furthermore, such repositories are typically restricted to specific data types (e.g. PPI or GRNs), precluding the integration of various data kinds that is critical to deepening our understanding of plant biology.

Several methods that extract gene functional information from literature have been developed to address these challenges. Predicate Logic for Predicting Protein Functions uses statistical methods to infer if a protein and a molecular term that describes protein function are semantically related (Taha et al. 2019). However, the method requires constructing complex statistical and linguistic models to link protein to function and only considers protein–function relationships. The EVEX database processes abstracts and full texts to identify regulatory relationships, posttranslational modifications, gene expression patterns, and other features of genes (Van Landeghem et al. 2013). However, the method also requires a manually constructed complex set of rules to extract and categorize the relationships, and the database has not been updated for a while. Another approach uses non-negative matrix factorization for feature reduction and then classifies the function of genes using K-nearest neighbor (Fodeh and Tiwari 2018). While the approach can reveal gene functions (e.g. gene A encodes a transcription factor), it does not reveal gene-gene relationships (e.g. gene A regulates gene B). STRING is a popular database that integrates protein–protein data, genomic features, co-expression, and text mining to build gene co-function networks (Szklarczyk et al. 2023). However, the text mining approach only identifies genes that are frequently mentioned together and cannot reveal the type of the relationship (e.g. interaction, regulation, activation) or identify relationships between genes and other entities (e.g. treatments, hormones). KnetMiner uses a rule-based approach to build knowledge graphs capturing relationships between various entities (Hassani-Pak et al. 2016). However, the rule-based system requires the integration of multiple heterogenous datasets (e.g. 12 types of data) (Hassani-Pak et al. 2016), making it difficult to include new data, species, and evidence types into the network. Furthermore, the database is gene-centric and does not allow searching the knowledge graph to understand how the different types of entities are related (e.g. traits and hormones).

In this paper, we aim to address the 2 fundamental challenges in gene function prediction: integrating the burgeoning information from scientific literature and using it to generate gold standard data for gene function prediction approaches. To achieve this, we seized the recent developments in Large Language Models (LLMs) to process over 71,000 research papers from leading journals in plant biology. Our approach excavated 4.8 million functional relationships between more than 2.7 million entities comprising gene products, metabolites, tissues, organs, and other biological components. The manual inspection of these relationships revealed not only their high accuracy and ability to even double the amount of functional information relative to the current coverage of gene regulatory networks. To provide access to these data, we constructed PlantConnectome, a user-friendly database containing a knowledge graph that can integrate information pertaining gene function, organ development, gene regulatory networks, protein–protein interactions, and other biological entities. PlantConnectome is available at https://plant.connectome.tools/.

## Results

### Meta-analysis of 71,136 paper abstracts

**Figure 1.**
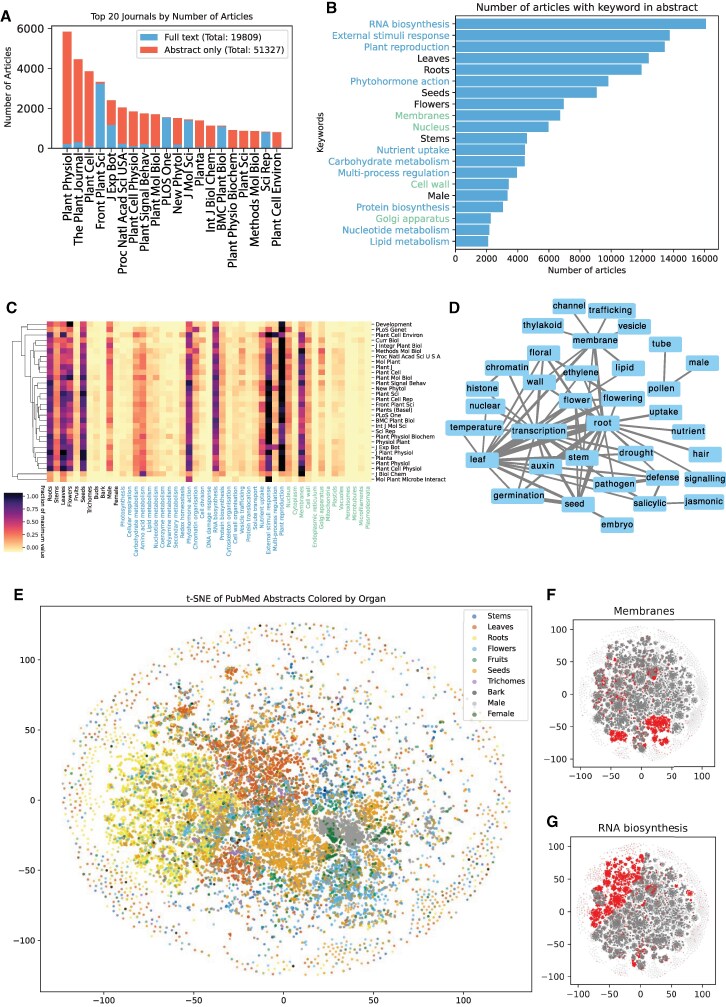
Meta-analysis of the 71,136 article abstracts. Meta-analysis of plant literature. **A)** Top 20 journals of the 71,136 articles analyzed in this study. The red and blue bars indicate the abstract-only or full-text articles, respectively. **B)** The number of abstracts (*x*-axis) with a given keyword (*y*-axis, color coded according to categories—black for organs/tissues/cells, blue for biological processes and green for subcellular compartments) of the analyzed papers. Each abstract can contain multiple keywords. **C)** Clustermap of top 30 journals (rows) and topics (columns). The colormap corresponds to the fraction of a maximum value found in a row (journal) across the 71,136 articles. The *x*-axis labels are color coded according to categories—black for organs/tissues/cells, blue for biological processes and green for subcellular compartments. **D)** Co-occurrence network of keywords in abstracts. Nodes represent keywords, while edges connect keywords found in at least 600 abstracts. Edge width is proportional to the number of abstract where any 2 keywords are co-mentioned. **E)** t-distributed stochastic neighbor embedding (t-SNE) visualization of the abstracts with a focus on plant organs. Each point represents an article, and the colors indicate the different organs. The plots for **F)** “membranes” and **G**) “RNA biosynthesis” are shown to the right.

To retrieve articles that focus on *Arabidopsis thaliana* genes and how these genes are related to other biological entities, we searched for articles that mention *Arabidopsis thaliana* and gene IDs in the abstracts (Supplementary Table S1). In total, 71,136 articles, of which 19,809 and 51,327 were accessible as full-text articles or abstracts only, respectively (Fig. 1A, list of all papers in Supplementary Table S2). The top 20 journals comprise Plant Physiology, the Plant Journal, and Plant Cell, for which most articles were not available for high-throughput download as full text (Fig. 1A, red bars). Conversely, the open access policies and the option to programmatically download the articles of the Frontiers in Plant Science, PLOS One, New Phytologist, BMC Plant Biology and Scientific Reports allowed us to download full-text articles from these journals.

To investigate whether the top 20 journals tend to publish specific topics, we determined the surveyed journals' discussion of cellular compartments, organs, and biological functions to assess their considered research topics. To this end, we defined a list of keywords pertaining to organs (e.g. roots = [root, hair, nodule, mycorrhizae]), biological processes (photosynthesis = [photosynthesis, photorespiration, photosystem]), and cellular compartments (e.g. nucleus = [nucleus, nucleolus, chromosome, nuclear pore]; Supplementary Table S3). We counted the number of these keywords in each abstract. Most journals did not show particular specificity for any topic, except Development (focus on reproduction, red cell), Journal of Biological Chemistry (membranes) and Molecular Plant-Microbe Interactions (external stimuli responses; Fig. 1, B and C). The most commonly studied organs were roots, leaves, flowers, and seeds, and the most studied pathways were phytohormone action (how hormones work), external stimuli response (how plants respond to the environment), RNA biosynthesis (how gene expression is regulated), and plant reproduction and most studied subcellular compartments were membranes, and nucleus (Fig. 1, B and C). Next, we investigated which keywords tend to co-occur in abstracts (Supplementary Table S4), which revealed, e.g. that pathogen research focuses on salicylic acid and transcriptional responses and uses leaves and roots as model organs (Fig. 1D).

To visualize the relationships among the abstracts, we generated a two-dimensional t-distributed Stochastic Neighbor Embedding (t-SNE) plot (Macosko et al. 2015), of the keyword counts. This technique allows us to represent high-dimensional data in a way that preserves local similarities between abstracts. We used a perplexity value of 40, which balances the attention between local and global aspects of the data, and ran 1,000 iterations to ensure convergence to a stable configuration (Supplementary Fig. S1 shows the influence of t-SNE parameters). The resulting plot provided an interpretable layout that highlighted clusters of abstracts with similar content or themes. The plots demonstrate clear groupings by biological processes (Supplementary Fig. S2), subcellular compartments (Supplementary Fig. S3), and organs (Supplementary Fig. S4), providing a bird's eye view of plant literature (Fig. 1E). For example, while articles describing male tissues (grey cluster, center right, Fig. 1E) also address “membranes” (Fig. 1F) and “RNA biosynthesis” (Fig. 1G), the majority of research on these topics is done in roots (yellow points) and leaves (dark orange points).

### Text mining research papers with large language model reveals 4,721,071 relationships between 2,386,814 entities

**Figure 2.**
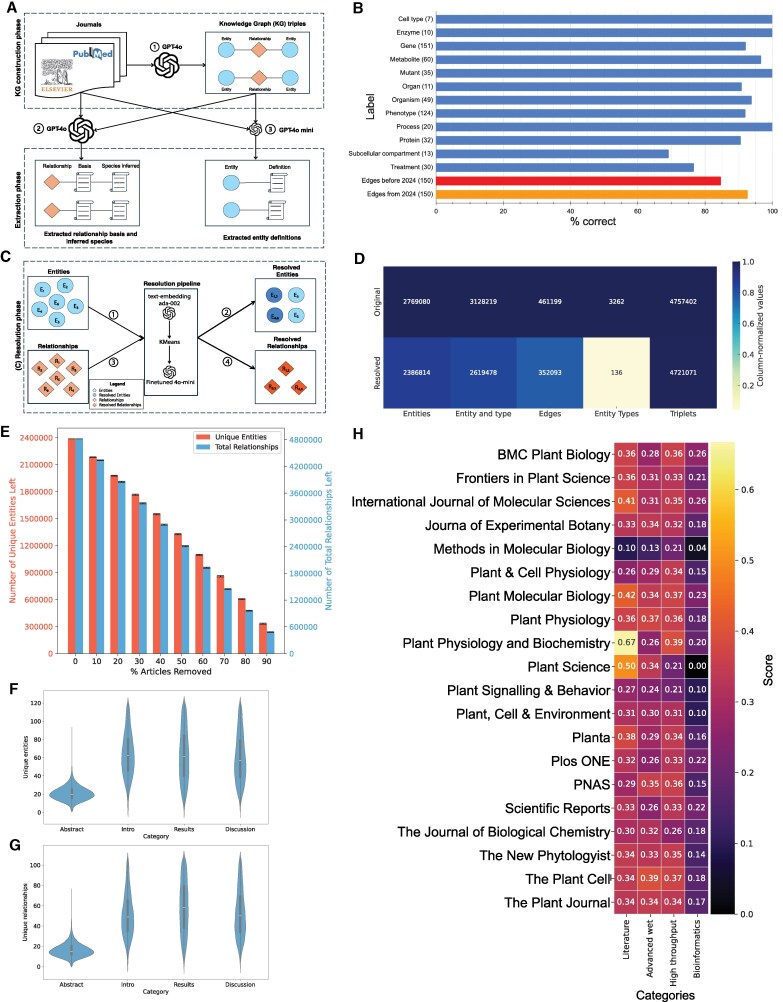
Evaluation of the plant knowledge graph. **A)** The pipeline to extract: 1. the knowledge graph from the literature, 2. species and relationship basis and 3. entity definitions. **B)** The percentage of correct entities (blue bars) and relationships (red and orange bars). The *x*-axis indicates the percentage of correct items inferred from manual curation. **C)** The pipeline to resolve redundant entities and relationships. **D)** The number of entities, entities and their types, edges, entity types and triplets (node, edge, node) in the original (top) and the resolved knowledge graph. **E)** The number of entities (red bars, left *y*-axis) and relationships (i.e. number of edges, blue bars, right *y*-axis) as a function of % articles removed (*x*-axis). The error bars represent the standard deviation. The data were generated by randomly removing a given percentage of articles 100 times. **F)** and **G)** The number of relationships and entities extracted from abstracts, introductions, results and discussions, respectively. Violin plots show the distribution of the number of unique entities per category after excluding statistical outliers. The distribution includes only values within the interquartile range (IQR) bounds, defined as Q1−1.5 × IQR to Q3 + 1.5 × IQR, where Q1 and Q3 are the 25th and 75th percentiles, respectively. Each plot reflects the density and spread of the non-outlier data, with the central width indicating concentration around the median. Outliers falling outside these bounds were excluded from the visualization. **H)** Average score depicting the diversity of methods extracted from the top 20 journals (Fig. 1A). The method categories include “literature” citations, “advanced wet” lab techniques, “high throughput” experiments and “bioinformatics”. A score of 1 indicates that a given journal has, on average, used all types of methods under one category, while a score of 0 indicates that no methods have been used. A method category is comprised of keywords. For example, “bioinformatics” is comprised of: “sequence”, “phylogenetic”, “genomic”, “alignment”, and “differential expression”. The code and keywords are available in Supplementary Data Set 1.

To extract information pertaining genes, metabolites, organs, environmental conditions, and other entities, we tasked OpenAI's Generative Pre-trained Transformer (GPT) models with identifying functional relationships between pairs of entities (e.g. “gene A”—interacts with—“gene B”; Fig. 2A, prompt 1 with GPT-4o in Supplementary Table S5) and also identifying the types of each entity (genes, metabolites, organs, treatments, others). The output of this analysis was a Knowledge Graph (KG), where nodes represent entities and edges represent relationships (e.g. “interacts with”, “regulates”, “causes”). To better understand which types of evidence underpin each relationship (e.g. “pull-down assay”, “co-expression analysis”), we also asked GPT-4o model to reveal the relationship basis and species the experiments were performed in (Fig. 2A, prompt 3 with GPT-4o in Supplementary Table S5). Finally, we tasked GPT-4o-mini model to annotate the extracted entities (e.g. “CESA”—is—“Cellulose Synthase A”; Fig. 2A, prompt 2 with GPT-4o-mini in Supplementary Table S5). The process yielded a large KG comprising 4,819,469 relationships between 2,771,008 entities (knowledge graph available from https://plant.connectome.tools/download).

Large language models are known to hallucinate and misunderstand the text, and to evaluate the accuracy of the identified entity types, we randomly selected 300 edges from the KG. We manually evaluated whether the identified entity types (e.g. “flavonol” type is “metabolite”) are correct, by comparing the entity types with their known biological function. Overall, we observed >90% accuracy in entity type classification, with the exception of “subcellular compartment” and “treatment” (Fig. 2B, Supplementary Table S6). The incorrectly classified “subcellular compartment” entity types comprised genomic features such as “distal enhancer elements”, and protein domains (“13 transmembrane [TM] helices”; Supplementary Table S6), while incorrect “treatment” types comprised methods and resources (e.g. “microsomal preparations”, “Genbank”).

To evaluate the accuracy of the edges, we manually compared them to the text from which they were extracted. Furthermore, since GPT-4 models have an October 2023 knowledge cutoff at the time of this analysis and could have been trained on the analyzed articles, we chose edges from articles published before 2024 and from 2024. Overall, we observed a high accuracy of 85% (before 2024, Fig. 2B red bar) and 93% (2024, orange bar), showing that the models can extract information from any text and not just regurgitate training data. The incorrect relationships typically misunderstood a hypothesis of the authors (source sentence: “*(results) made us wonder whether this tissue-specific polarization of PIN-formed (PINs) is conserved in other bryophytes*”), incorrectly producing a fact edge (“*tissue-specific polarization of PINs conserved in other bryophytes*”; Supplementary Table S6).

Manual inspection of the knowledge graph revealed that a number of entities (e.g. *Arabidopsis thaliana*, *A. thaliana, thale cress*), entity types (e.g. gene group, gene family), and edges (e.g. controls, regulates) are synonymous. These variations arise from inconsistent naming conventions, capitalization, pluralization, and spelling differences between articles. Such redundancy inflates the number of unique elements in the graph, obscures true connectivity, and complicates downstream analyses. To address this, we implemented an automated resolution protocol that utilizes semantic embedding, clustering, and large language models to enhance overall cohesion and reduce unnecessary repetition within the PlantConnectome (Fig. 2C, Supplementary Fig. S5). The resolved graph contained 86.2% of entities, 83.74% entity and entity type combinations, 76.34% relationship types, 4.17% entity types, and 99.24% of triples (node, edge, node) of the original knowledge graph (Fig. 2D). The high number of retained triples indicates that while the entities and relationships tend to be redundant, many of the triples (entity-relationship-entity) are unique and non-redundant. We observed a high accuracy of the resolved relationships (87.33%, Supplementary Table S7), entity types (91.33%, Supplementary Table S8), and triples (79.33%, Supplementary Table S9).

To investigate the relationship between the number of articles and the number of identified entities and relationships, we randomly removed 10–90% of articles 100 times and recounted the number of retrieved items. Overall, we observed a linear relationship between the number of articles and the retrieved data (Fig. 2E), indicating that more articles would expand the KG further. The amount of information extracted from the introduction (median 50 and 63 relationships and entities extracted, Fig. 2, F and G), results (59, 63), and discussion (47, 57) was higher than from abstracts (16, 21). Thus, increasing the number of full text articles would further expand the KG.

Finally, we investigated which types of evidence are present in the top 20 journals. We categorized the evidence into “literature” (article citing findings from other articles), “advanced wet” (article using advanced experimental approaches, such as pull-down, transgenic lines), “high throughput” (evidence based on, e.g. RNA-seq analysis, differential gene expression) and “bioinformatics” (evidence based on sequence alignment, phylogenetic tree, Supplementary Data Set 1 contains the used code and keywords). Overall, the evidence profiles of the different journals were similar, with Plant Cell and Plant Physiology on average using more advanced wet lab methods (advanced wet > 0.35, Fig. 2H). Plant Science, Plant Physiology, and Biochemistry contain extensive literature-based evidence. In contrast, Methods in Molecular Biology which focuses typically on one method contained the least diversity of the used evidence types.

### Properties of the connectome network

**Figure 3.**
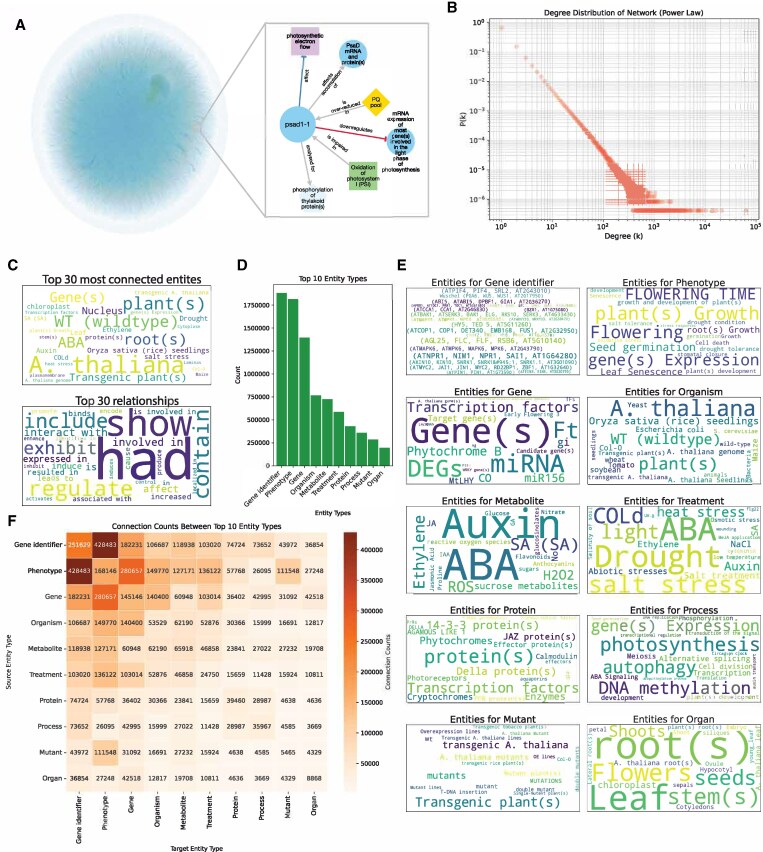
Properties of the Connectome knowledge graph. **A)** Gephi visualization of the Connectome knowledge graph colored by node degrees. The ForceAtlas 2 algorithm was run until convergence with a stronger gravity law and a scaling factor of 0.5 to separate the graph's nodes. Light blue nodes represent nodes with the fewest degrees, and light green nodes are the nodes with the highest degrees. **B)** The degree distribution of the knowledge graph, where nodes represent entities and edges represent relationships between these entities. The *x*-axis (degree (k)) represents the number of connections (relationships) each entity has. In contrast, the *y*-axis (frequency P(k)) shows the frequency of an entity having exactly k connections. **C)** Top most connected entities (top) and observed relationship (bottom) types. The size of the lettering is proportional to the number of relationships. **D)** Top 10 entity types, with entities (*x*-axis) and their numbers (*y*-axis). **E)** Top 20 entities found in the top 10 entity types. **F)** The number of edges between the top 10 entity types.

We used the KG to construct the Connectome network, and visual inspection of the whole graph revealed cluster-like structures of densely connected entities (Fig. 3A). Certain networks, such as protein–protein interactions, display scale-free behavior, where most nodes have few connections, and few nodes/entities have many connections (Broido and Clauset 2019). To investigate whether the Connectome is scale-free, we constructed a scatterplot of its log-transformed node frequency (*p(k)*) and node degree (*k*) (Fig. 3B). The points formed a line with a negative slope, indicating a typical power law distribution (Mutwil et al. 2010), indicating that most entities have a few relationships, while a small number of entities act as hubs with a large number of connections.

We next investigated which entities and relationships are most important in the KG. General entities, such as “A. thaliana” and “plants”, and general relationships, such as “had”, “show” were most frequently observed (Fig. 3C), but we also observed more specific entities (e.g. auxin, abscisic acid [ABA]) and relationships (regulate, interacts with). The most common entity types comprised “gene identifier” (genes with an identifier we could detect) and “phenotype”, in line with our selecting articles containing gene names and “*Arabidopsis thaliana*” (Fig. 3D, Supplementary Table S1). A closer look at the entity types revealed the most common entities for gene identifiers (e.g. elongated hypocotyl 5, flowering locus C, nonexpressor of pathogenesis-related genes), phenotypes (growth, flowering, germination, gene expression), genes (comprising generic gene(s), micro ribonucleic acid, differentially expressed genes), organism (A. thaliana), metabolite (ABA, auxin, ethylene, reactive oxygen species, salicylic acid [SA)], treatment (drought, ABA, salt stress), protein (general transcription factors, phytochromes, protein(s)), process (photosynthesis, deoxyribonucleic acid [DNA] methylation, autophagy), mutants (general “transgenic plants”) and organ (root, leaf; Fig. 3E). Finally, we investigated how often the different entity types are connected in the network. We observed most connections between “gene identifier”−”phenotype”, “mutant”−”phenotype” and “gene”−“treatment” (Fig. 3F), likely reflecting the typical function studies that characterize genes in terms of mutant phenotypes and responses to various treatments.

### Evaluation of the coverage and accuracy of the PlantConnectome

Our main motivation in this study was to expand the amount of the gold standard data capturing experimentally-verified gene functions. In total, we identified 82,800 (directional, where gene order a → b is kept) and 69,071 (unidirectional, where order is disregarded) unique gene-gene relationships (https://plant.connectome.tools/download). We, therefore, investigated the overlap of relationships in our KG with data provided in the public repositories.

**Figure 4.**
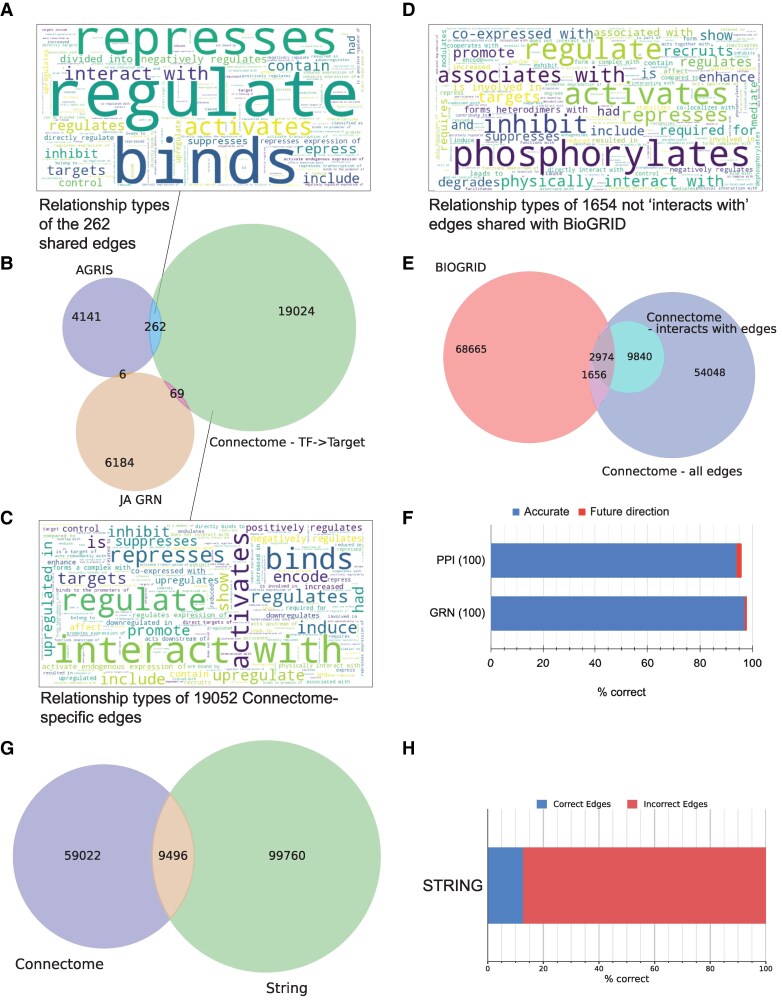
Evaluation of the gene regulatory and protein-protein interaction networks identified by the Connectome. **A)** Word cloud of the 206 relationship types shared between AGRIS and the Connectome. **B)** Venn diagram showing the overlap between AGRIS, the Connectome and the Jasmonic Acid Gene Regulatory Network (GRN). The numbers indicate edges found between transcription factors and putative target genes. **C)** Word cloud of the 14736 Connectome-specific edges. **D)**  *Word cloud of the 1,369 edges shared between BIOGRID and the Connectome. The edges do not belong to the “interacts with group”.*  **E)** The Venn diagram showing the overlap between BIOGRID and the Connectome. All gene-gene edges in Connectome are indicated with a blue circle, while the lighter inner circle shows genes connected by “interacts with” relationships. **F)** Accuracy evaluation of the protein-protein interaction (PPI) and gene regulatory network (GRN) edges that are specific to the Connectome. The blue and red fields indicate correctly inferred or “future direction” relationships, respectively. **G)** Venn diagram showing the overlap between Connectome (blue circle) and STRING (green circle). **H)** Accuracy of the STRING edges.

To compare the coverage and the accuracy of GRNs, we obtained the *Arabidopsis thaliana* gene regulatory network from AGRIS (https://agris-knowledgebase.org/downloads.html, updated March 2019) (Yilmaz et al. 2011), comprising 4,409 confirmed transcription factor → target edges. We also identified 6,259 edges from a study investigating responses to jasmonic acid (Zander et al. 2020). Next, we identified 19,383 directional transcription factor → target edges in the Connectome (https://plant.connectome.tools/download). The edges shared between AGRIS and the Connectome comprised of “regulate”, “binds”, “activates”, “targets”, demonstrating the Connectome's ability to identify the various functions of transcription factors (Fig. 4A). However, we observed a very minor overlap (e.g. 262 edges between AGRIS and the Connectome) between the three GRNs (Fig. 4B), indicating the high dissimilarity between the GRNs. We identified 19,052 Connectome-specific edges between transcription factors and target genes, and the most frequent association was “regulate”, “binds”, “activates”, “represses”, and others (Fig. 4C). This indicates that the Connectome transcription factor networks seamlessly integrate PPIs and GRN networks.

Furthermore, we compared the protein–protein (PPI) network from BioGRID to the Connectome's unidirectional edges. We found 1,654 edges shared between BioGRID and the Connectome that were not of class “interacts with, forms a complex with” (or similar), and found that the edges comprised of types such as “phosphorylates”, “associates with”, but also “represses” (Fig. 4D). This indicates that the Connectome can provide additional nuances to the PPIs. Overall, we also found a relatively poor overlap between BioGRID and Connectome, with only 2,974 “interacts with” edges shared (Fig. 4E), and the large majority of edges being specific to each database.

To validate the accuracy of the Connectome-specific edges, we randomly sampled 100 GRN (out of 14,736) and 100 PPI (out of 8,234) edges (code in Supplementary Data S1), and inspected their accuracy by reading the corresponding text (Supplementary Table S10). Overall, we observed 97% and 94% accuracy for the GRN and PPI networks, respectively (Fig. 4F). In agreement with the misclassified entities and relationships (Fig. 2B), GPT-4o confused future directions (e.g. sentence in the manuscript: “*In this regard, it will be interesting to investigate whether FUSCA3 (FUS3) interacts with insulin-dependent diabetes susceptibility 8 (IDD8) through the fourth zinc finger (ZF) domain.*”) as a fact (*GPT-4o edge “FUS3 interacts with IDD8*”; Supplementary Table S10). These results indicate Connectome's valuable companionship and alternative role to not only AGRIS but also BioGRID.

Lastly, to compare our text-mining pipeline with the state of the art, we extracted high-confidence edges from the STRING database (text mining score score >700; Holtzapple et al. 2020; Szklarczyk et al. 2023) and selected common AGI identifiers between STRING and our Connectome. This identified 109,256 unidirectional edges in STRING, of which only 9,495 (8.7%) are shared between STRING and Connectome (Fig. 4G). We validated the STRING database by randomly sampling and manually evaluating 150 edges (as done in Fig. 4H), which revealed a low 12.7% accuracy for the STRING-specific edges (Supplementary Table S11).

### Features of PlantConnectome

To provide access to the Connectome, we constructed a dedicated database (https://plant.connectome.tools/), which offers numerous methods of searching for genes, metabolites, organs, and other entities by terms, author names, and PubMed IDs, alongside a catalogue page (accessible under the https://plant.connectome.tools/catalogue) listing all entities in the database. An entire information page is also provided for each entity in the connectome, containing its definitions (e.g. CESA: “A large family of genes encoding cellulose synthases and related enzymes”) and source article.

**Figure 5.**
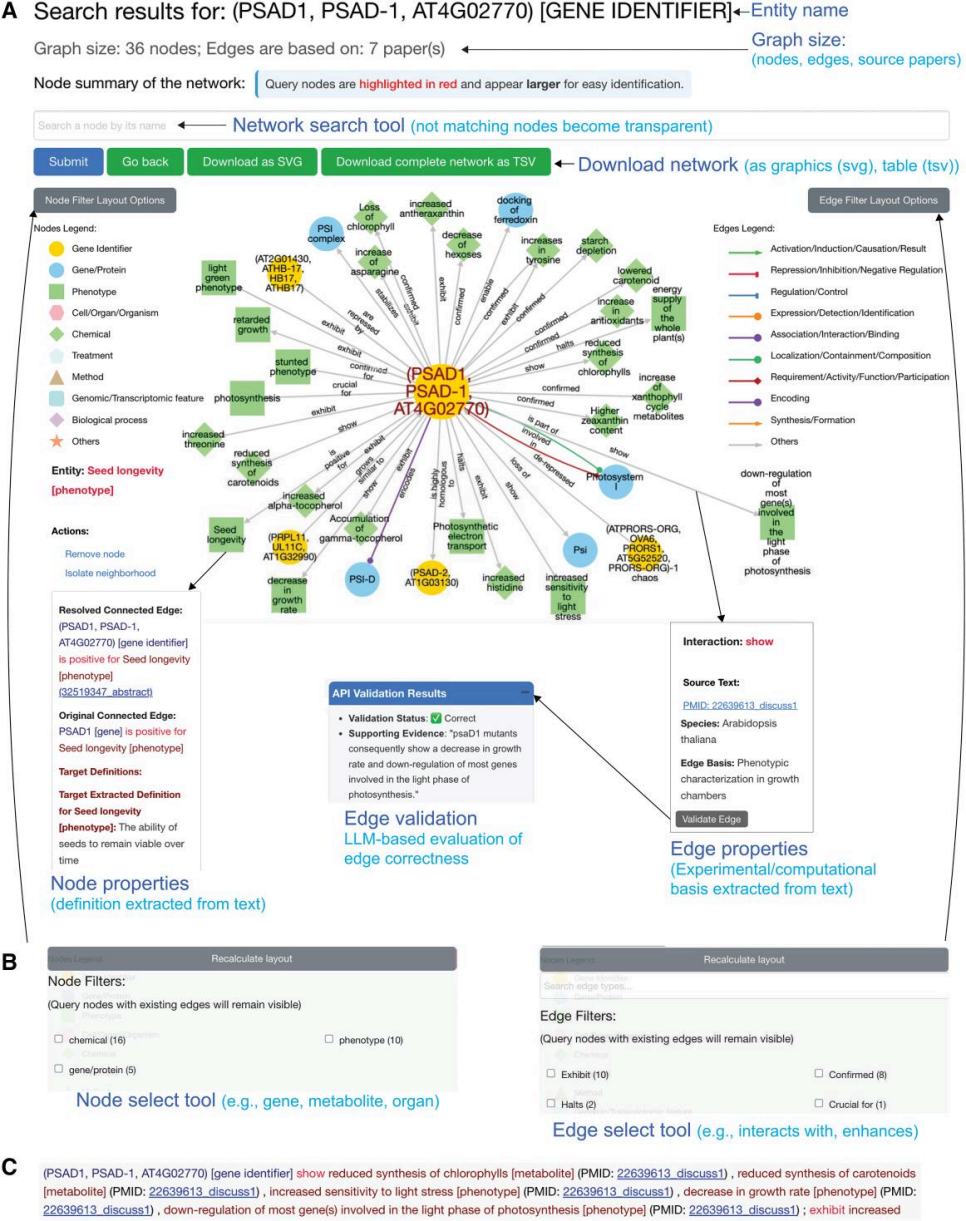
Outline of the Connectome entity page. **A)** The top of the page contains the entity name and the graph size, comprising the number of nodes (entities) and papers that are used to build the current graph. Note that the graph size changes dynamically when the user adjusts the node and edge selection. The graph is visualized as an interactive network implemented in cytoscape.js, where the nodes can be moved around, and the different elements can be clicked on to open tooltips that display additional information. Query nodes are larger and labeled with red text. The entities in the network can be searched by writing the entity's name and clicking submit (blue button). The network can be downloaded as a vector graphic image and tab-delimited table. Clicking on the edges displays which type of evidence underpin an edge, while the “Validate edge” button tasks a LLM with confirming the validity of the edge. The LLM will also show the sentences that underpin an edge. **B)** The users can select the entity types (e.g. genes/proteins, metabolites) and edge types (e.g. regulates, interacts with) of interest by clicking on the Node and Edge select tools. **C)** The text summary provides an organized, textual representation of the network and PubMed IDs that underpin each edge. The text summary is dynamic and responds to the node and edge selection performed by the user.

To detail PlantConnectome's search result page, we performed a standard query with the gene “Photosystem I Subunit D-1 *(Psad1)*” (“Mutant affecting photosystem I complex in plants”, https://plant.connectome.tools/normal/psad1), which is involved in the formation of photosystem I (Ihnatowicz et al. 2004). We selected the first hit, which took us to the entity's landing page. The page displays the number of nodes in the knowledge graph and the number of papers used to construct the KG, together with the extracted definitions of the entity (Fig. 5A).

The knowledge graph is represented as an interactive network, depicting the various relationships the search query shares with other entities in the database (Fig. 5A). Upon clicking on a node, the user is provided with a “Node properties” tooltip displaying the node's definitions, and a set of options enabling the removal of the node or isolation of the node's neighborhood. Note that the nodes containing the query term remain as long as they are connected to other entities to ensure that relationships between the query and other categories can always be viewed. Clicking on an edge opens an “Edge properties” tooltip that displays the PubMed ID underpinning an edge and shows the experimental basis (if available) of the edge. Users can select the node and relationship types by clicking on the “Node select” and “Edge select” tools and thus focus on the entity and relationship types of interest (Fig. 5B). The current network view can be downloaded as an image (SVG) or as a tab-delimited table, ready for further processing in, e.g. Cytoscape. The network is also available as a text summary (Fig. 5C) and a table, where clicking on a given PubMed ID will prompt a popup containing the corresponding abstract. The text summary adjusts its content to the selections specified in the “Node select” and “Edge select” tools.

Since the knowledge graph is not 100% accurate, we provide two options for the users to validate the information. First, the user can click on the edge and select the “Source text” link, which will display the source text used to generate the edge. The source text can be inspected by the reader. Second, the user can click the “Validate edge” button, which will use an LLM to compare the edge with the source text and approve or reject an edge (Fig. 5A). The source text links also indicate which part of the article the text originated from. For example, link 37008510_intro shows that the text and the resulting edge originated from the introduction of an article with PubMed ID 37008510. Furthermore, clicking on nodes and edges also shows the original and resolved entities and relationships, respectively.

Finally, PlantConnectome enables users to perform searches through an API, which returns a JSON object containing relevant network and functional information, extending its functionality to bioinformaticians who desire programmatic access to our database. As an example, an alias search on the PSAD1 gene may be performed by accessing the URL “https://plant.connectome.tools/api/normal/psad1”.

### Examples of how to use PlantConnectome

We provide 3 case studies, comprising protein complexes, gene regulatory networks, and stress responses, to demonstrate how the Connectome can be used to rapidly summarize available knowledge.

#### Example 1: Translocon on the outer chloroplast membrane complex

To exemplify how the Connectome can be used to study protein–protein interactions and identify protein complexes, we selected the first hit found by “TOC complex” as a query for the “gene/word” search (https://plant.connectome.tools/normal/toc complex). This identified a knowledge graph containing 104 nodes based on 58 papers. We then narrowed down the graph to nodes representing genes with “gene identifier” using the “Node filter layout” tool, and “Consists of” with the “Edge filter layout” tool, resulting in 11 nodes based on 6 papers.

**Figure 6.**
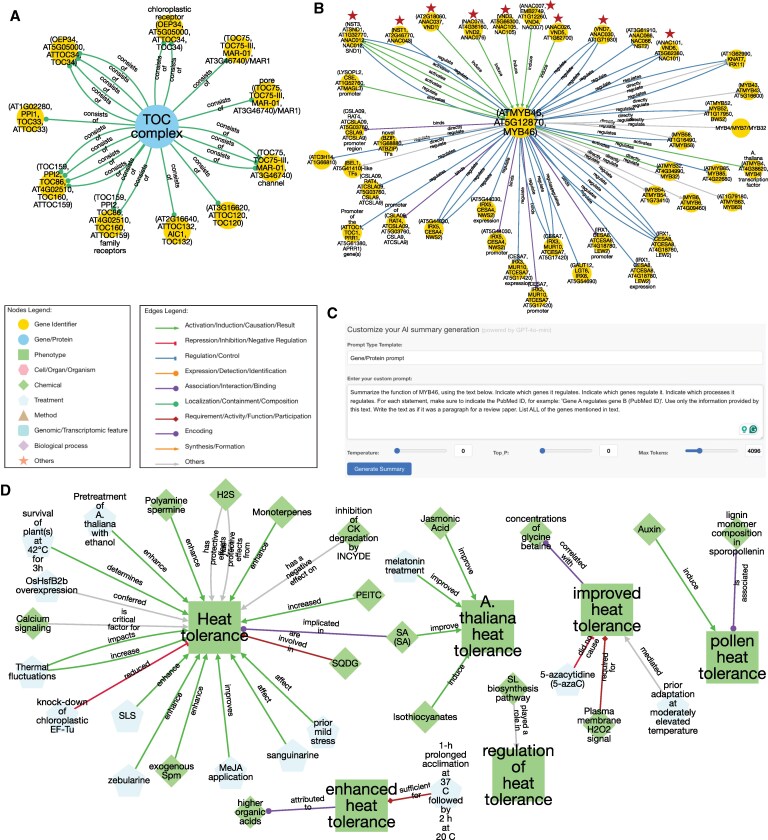
Usage examples of the Connectome database. **A)** “Gene identifier” neighborhood of “TOC complex” found by gene/word search. Nodes represent gene/protein entities, while edges depict relationships. The node and edge legend indicate the different types of entities and relationship types, respectively. **B)** Gene identifier neighborhood of *MYB46*. Transcription factors regulating MYB46 are indicated with red stars. **C)** AI summary generation tool. Custom prompt used was indicated in the text box. **D)** Neighborhood of “heat tolerance”-containing entities. The nodes were selected to only comprise of “chemical” and “treatment” entity type.

Translocase complexes on the outer and inner envelope membranes (TOC and Translocon on the outer chloroplast membrane [TIC], respectively) are used to import proteins into the chloroplast (Stengel et al. 2009). We compared the TOC75 graph to a review on the translocase complexes (Richardson and Schnell 2020), which revealed known TOC75 interactions such as *TOC 22, 34*, *159*, and *TIC236* (Fig. 6A). The associated nodes also provide additional genes relevant for TOC75 function, such as dek5 mutant, which is reducing the levels of TOC75 (experimental organism: maize, evidence: proteomics analysis and immunoblotting of chloroplast envelope proteins) (Zhang et al. 2019), and chaperone heat shock protein 90C (HSP90C) that interacts with many of the translocon proteins (experimental evidence: Coprecipitation experiments with protein import components) (Inoue et al. 2013). Thus, the Connectome allows a rapid elucidation of protein–protein interactions and subunits of protein complexes and provides source literature and evidence types supporting these interactions.

#### Example 2: Secondary cell wall master regulator

To demonstrate how our database can be used to study gene regulatory networks, we selected the secondary cell wall biosynthesis regulator, MYB domain protein 46 (*MYB46)*. To this end, we entered “MYB46”, used the “gene/word” search and selected the first entity. The initial network contained 283 nodes based on 108 papers, but to focus on genes, we selected nodes with “gene identifiers”, which reduced the network to 66 nodes based on 51 papers. Since MYB46 is a transcription factor, we selected edges capturing typical gene regulatory relationships (“regulate”,“activates”,“directly regulates”,“binds”,“regulates”,“induce”) from the “Edge filter” menu, and arrived at a network comprising 40 nodes based on 23 papers (Fig. 6B). A literature search on the gene regulatory network underlying secondary cell wall formation revealed a large overlap between the output of the Connectome and the figure in the review article (Xiao et al. 2021).

Genes regulated by *MYB46* included the secondary cell wall cellulose synthases (*CESA4,7,8*), and a panel of downstream transcription factors (*MYB4, 7, 32, 43, 52, 54, 58, 63, 85*). Genes regulating *MYB46* comprised transcription factor staphylococcus nuclease and tudor domain containing 1 (*SND1)* (Fig. 6B). Interestingly, our Connectome, but not the review, identified vascular related NAC-domain proteins (*VND1-5, 7)* and nac secondary wall thickening promoting factor (*NST1-3)* (evidence for NST2: Direct target analysis using the estrogen-inducible system) (Zhong et al. 2008) and *VNDs* (evidence: Quantitative PCR analysis of *VND1* overexpressors showing induction of *MYB46* gene expression) (Zhou et al. 2014)as regulators of *MYB46*. On the other hand, our Connectome missed downstream *MYB20, 42, 75, 103* (Xiao et al. 2021).

Since the knowledge graph is summarized in a human- and machine-readable format, we investigated whether ChatGPT can generate publication-ready passages. To this end, the network summary (exemplified in Fig. 5C) can be analyzed by the AI summary generation tool (Fig. 6C), which allows the user to summarize the current network as text. For example, the MYB46 network in Fig. 6B was analyzed with this prompt:

*Summarize the function of MYB46, using the text below. Indicate which genes it regulates. Indicate which genes regulate it. Indicate which processes it regulates. For each statement, make sure to indicate the PubMed ID, for example: “Gene A regulates gene B (PubMed ID)”. Use only the information provided by this text. Write the text as if it was a paragraph for a review paper. List ALL of the genes mentioned in text.*

The output is: *MYB46 (ATMYB46, AT5G12870) is a transcription factor that plays a crucial role in the regulation of various genes involved in plant development and secondary cell wall formation. It directly regulates MYB63 (AT1G79180) (PMID: 24009617_intro3), BEL1-like transcription factors (AT5G41410) (PMID: 24009617_intro3), KNOTTED-Like From Arabidopsis thaliana 7 (KNAT7) (AT1G62990) (PMID: 24009617_intro3), MYB43 (ATMYB43, AT5G16600) (PMID: 24009617_intro3), MYB58 (AT1G16490) (PMID: 24009617_intro3), MYB54 (ATMYB54, AT1G73410) (PMID: 24009617_intro3), MYB6 (ATMYB6, AT4G09460) (PMID: 24009617_intro3), and MYB4/MYB7/MYB32 (PMID: 24009617_intro3). Additionally, MYB46 binds to the promoters of irregular xylem 1 (IRX1) (AT4G18780), CESA8 (ATCESA8), and LEW2 (PMID: 31191566_discuss2), as well as the promoter of cellulose synthase like A9 (CSLA09) (AT5G03760) (PMID: 31554868_intro) and the promoter of CESA7 (AT5G17420) (PMID: 31191566_discuss2). It also regulates the expression of IRX1 (PMID: 31191566_discuss2), CESA8 (PMID: 26011122_abstract), and the expression of genes such as AT5G44030 (IRX5), CESA4, and nuclear receptor binding SET domain protein 2 (NWS2) (PMID: 37047127_discuss). MYB46 is activated by NST3 (AT1G32770), secondary wall-associated nac domain protein 1 (ATSND1), ANAC012, NAC012, and SND1 (PMID: 33537043_intro), and it is also induced by nac domain containing protein 76 (NAC076) (AT4G36160), vascular-related nac domain protein (VND2), nac domain containing protein (ANAC076) (PMID: 25148240_results2), ANAC037 (AT2G18060), VND1 (PMID: 25148240_results2), ANAC007 (AT1G12260), VND4 (PMID: 25148240_results2), and ANAC026 (AT1G62700) (PMID: 25148240_results2). Furthermore, it is regulated by ANAC101 (VND6, AT5G62380) (PMID: 35885964_intro2) and VND7 (ANAC030, AT1G71930) (PMID: 33267872_intro). Overall, MYB46 is a key regulator in the transcriptional network governing secondary cell wall biosynthesis and related developmental processes*.

Importantly, since we asked the large language model to only use the existing PubMed IDs and only utilize the information provided by the network, it does not hallucinate non-existing references or information. The provided “AI summary generation” tool allows the users to utilize premade prompts or write their own to extract specific information. However, the users need to keep in mind that our tool reports all extracted relationships, including those mentioned in article Introductions. Since Introductions typically refer to prior work, it is likely that some of the extracted edges might discuss previous findings. Thus, we recommend the users to verify all edges to determine whether a relationship stems from the current study or earlier literature. This could be done by utilizing the “Source text” link or “Validate edge” button, which are found when inspecting an edge.

#### Example 3: Heat stress

To demonstrate how the Connectome can be used to study entities that are not necessarily genes, we investigated treatments, chemicals, hormones, and metabolites that can affect heat tolerance. We searched for “heat tolerance” with “gene/word” search, and selected “heat tolerance”, “A. thaliana heat tolerance”, “enhanced heat tolerance”, “regulation of heat tolerance”, “improved heat tolerance”, “pollen heat tolerance”. The resulting large knowledge graph comprised 454 nodes from 263 papers, which is expected as heat stress is one of the most studied abiotic stresses in plants (Koh et al. 2024). We further focused the search by selecting “Chemical” and “Treatment” in the “Node filter” tool, which shrank the graph to 37 nodes based on 29 papers (Fig. 6D). As generated by the AI summary, notable metabolites include salicylic acid (SA) (PMID: 22378947, mentioned in discussion), lignin monomer composition in sporopollenin (37691288, results), monoterpenes (29197862, abstract), hydrogen sulfide (H2S) (30283480 introduction; 28992305 introduction), calcium signaling (32527735 abstract), sulfoquinovosyldiacylglycerols (SQDG) (37970465 discussion), polyamine spermine (31484414 discussion), organic acids (28158841 discussion), glycine betaine (9839462 abstract), isothiocyanates (26236322 introduction), and phenethyl isothiocyanates (PEITC) (25657654 introduction). Hormones such as jasmonic acid (32825569 introduction) and auxin (28574629 introduction) also play an important role in heat tolerance. Various treatments that enhance heat tolerance include thermal fluctuations (33119606 results), ethanol pretreatment (35729482 abstract), SLS (38279328 discussion), zebularine (33091182 discussion), melatonin treatment (27047496 discussion), Os08g0546800(OsHsfB2b) overexpression (36267847 discussion), methyl jasmonic acid (MeJA) application (37445710 discussion), and prior mild stress (10760238 abstract). Additionally, the inhibition of cytokinin (CK) degradation by INCYDE (32133021 discussion) negatively affects heat tolerance, while the knock-down of chloroplastic Elongation Factor Thermo Unstable (EF-Tu) (30915096 results) reduces heat tolerance. To conclude, the dynamic selection option of edge types in the network enables scrutinizing different relationship types between the entities found in PlantConnectome, and goes beyond gene-centric analyses.

## Discussion

We have illustrated GPT's text mining capacities in the context of scientific literature, processing over 71,000 research abstracts at a moderate cost (∼5,000 USD) and harvesting invaluable functional information therein. GPT could extract key entities and relationships from research paper abstracts with high accuracy (Figs. 2B and 4F) and few prompts (Supplementary Table S5). The amount of functional information excavated from the abstracts and full-text articles increased the amount of machine-readable data, as demonstrated by our gene regulatory networks that nearly tripled the available data (Fig. 4B). Moreover, PlantConnectome overcomes the limitations of typical databases that employ only one data type, as it draws upon numerous data sources in establishing gene functions, organ development, gene regulatory networks, protein–protein interactions, and other phenomena, all in a user-friendly manner.

Our evaluation has shown that PlantConnectome is not only comprehensive and accurate but also complementary to existing databases (Fig. 4). Comparing PlantConnectome's gene regulatory networks against AGRIS and its protein–protein interaction networks against BioGRID demonstrates that PlantConnectome's retrieved networks do not largely overlap with these reference databases. Rather, the GPT-extracted networks complement them, showing the effectiveness of our text-mining approach in utilizing the vast amount of literature that has not been captured by manual curation. The accuracy discrepancy between the Connectome (>93%) and STRING (12.7%) is likely due to our Connectome taking advantage of the natural language understanding of LLMs. Conversely, STRING's text mining algorithm only requires two genes to be mentioned in an abstract to be linked, which often introduces high number of false positives (Supplementary Table S11).

However, GPT's outputs are not entirely accurate and still warrant manual verification, as GPT-4o models have a tendency to misidentify entities and relationships (Figs. 2B and 4E), which is perhaps attributable to the varying language and content of the >71,000 processed articles. The correction of errors may be carried out by fine-tuning the models with manually curated examples containing the expected output (as, for instance, that found in Supplementary Table S6). Furthermore, our tool reports all of the found relationships, regardless if a relationship was demonstrated in a paper. For example, relationships extracted from Introduction are likely to report previous findings reported in past literature. Thus, the users of the Connectome are encouraged to click on nodes and edges to further validate these entities” accuracy and ensure whether a given relationship is reported in the current or past article.

In conclusion, PlantConnectome is an innovative tool, combining the power of a state-of-the-art language model with the comprehensive information embedded in a massive collection of research articles. The tool offers an efficient and diversified way to retrieve information for genes, metabolites, tissues, organs, and other biological components. The potential applications of PlantConnectome are wide-ranging and extend beyond those we have highlighted in this article. Furthermore, since we only analyzed articles mentioning *Arabidopsis thaliana* and its genes, the inclusion of all plant scientific literature together with the inclusion of more full-text papers is bound to increase the completeness of the knowledge graph, help us stay up to date with the plant literature, and provide gold standard data for gene function prediction studies. We anticipate that PlantConnectome will become a valuable resource for the plant science community to facilitate various research activities, from a preliminary investigation of gene functions to an in-depth study of a particular biological process.

## Materials and methods

### Retrieval of articles

Using BioPython version 1.81, we downloaded papers containing Arabidopsis thaliana species name and gene identifiers (e.g. At4g32410). For each gene identifier, we also searched with gene aliases (e.g. CESA1, RSW1) retrieved from www.arabidopsis.org (Supplementary Table S1). The NCBI query was the following: query = f'(Arabidopsis thaliana[Title/Abstract] AND {query_term}[tw]) OR (Arabidopsis[Title/Abstract] AND {query_term}[tw]) OR (Thale cress[Title/Abstract] AND {query_term}[tw] OR (Mouse ear cress[Title/Abstract] AND {query_term}[tw] OR (Mouse-ear cress[Title/Abstract] AND {query_term}[tw])’. Query_term are the genes in Supplementary Table S1, and other alternative names of Arabidopsis were included in the search. The code to perform this analysis is available in GitHub in Supplementary Data Set 1.

### Large language model analysis of articles

We used GPT4 models to extract entities and relationships (GPT-4o), entity definitions (GPT-4o-mini) and to identify the species that the article uses as a model (GPT-4o). Furthermore, for each entity relationship, we asked GPT-4o to identify the evidence (e.g. yeast two-hybrid, bioinformatic prediction) underpinning the relationship. We iterated over several prompts to arrive at prompts that yielded consistently accurate results on selected papers. In total, 71,136 articles were processed using OpenAI's batch API (Application Programming Interface; https://openai.com/api/pricing/). The code to perform this analysis is available as Supplementary Data Set 1.

### Entity type resolution

We based our entity type resolution on OpenAI's o3-mini, which we used to iteratively resolve less common types that are semantically similar to more frequent ones. We assigned the top 30 most common types as our reference set to preserve their integrity. Next, we looped through the remaining 3,231 types and calculated the 10 closest reference entity types using the cosine similarity of pre-calculated text embeddings from OpenAI's text-embedding-ada-002 model. We then submitted each of the remaining types and corresponding 10 closest reference types to o3-mini for processing; the type either resolved or was added to our reference set. This process produced a final set of 136 entity types, which we used in the downstream resolution of entities and edges. The code to perform entity type, entity, and relationship resolution are available at https://github.com/manojitharaju016/plant_kg_disambiguation.

### Entity resolution

We designed and implemented a pipeline (Supplementary Fig. S5) for resolving nodes, where we embed each node name, its type, and its definition into vectors of length 1536 with OpenAI's text-embedding-ada-002 model. We iteratively clustered the embeddings with k-means until the cluster size was less than or equal to 30; capping at 30 was done to fit the clusters into the limited context window of the LLM that we utilized for obtaining fine-grained clusters. Next, we fed the k-means clusters along with specific instructions (Supplementary Table S5) to OpenAI's o3-mini model, which outputted sub-clusters with a representative chosen for each sub-cluster. We again used the same model with a different set of instructions to validate its own clusters (Supplementary Table S5); the model outputs whether a cluster is valid or not, reasoning with an explanation of why the cluster is valid or invalid, and if invalid, gives the corrected sub-clusters. We utilized the corrected sub-clusters as ground-truths to fine-tune OpenAI's cost-optimized LLM, the GPT-4o model, on a subset of around 1000 input–output examples. The fine-tuning step was aimed at substantially reducing costs while maintaining performance comparable to advanced reasoning LLMs such as o3-mini, which otherwise incur substantially higher expenses. Finally, we leverage the fine-tuned 4o-mini model to obtain the sub-clusters.

### Relationship resolution

We reused the fine-tuned model for relationship resolution of k-means clustered relationship embeddings. We calculated embeddings using the source and target entity type, in the format; “[source type] relationship [target source”], to closely resemble the format used in training of the fine-tuned model. Finally, we also disambiguated the knowledge graph programmatically. To disambiguate relationships (e.g. “caused”, “cause”, “causes”) and entities (e.g. “Arabidopsis plants”, “Arabidopsis”, “Arabidopsis thaliana”), we identified the top 100 most common relationships and entities and devised a rule-based method to map the various synonyms or variations to a canonical form. Passive edges (e.g. “is regulated by”) were converted to active form (“regulates”). Entities that differed by casing (e.g. “Genes”, “genes”) were represented by one canonical form.

### API validation of entity–entity relationships and summary generation

To validate the accuracy of entity–entity relationships, we implemented a feature that allows users to leverage the GPT-4o-mini model via the OpenAI API (https://platform.openai.com/docs/overview). For each interaction, the original source text chunk is provided as context, and the model is prompted to evaluate whether the described relationship accurately reflects the content. Using the same pipeline, PlantConnectome also supports scientific review generation based on the summarized network data. Prompts are tailored for each entity type and the author_search page. Full prompt templates are provided in Supplementary Table S5.

### Construction of PlantConnectome database

The PlantConnectome is hosted on a Google Cloud server. The backend was implemented using the Python framework Flask and the Python packages networkx version 3.1, pickle version 3.11.4, json version 3.11.4, and regex version 3.11.4. We used JavaScript dependencies jQuery v3.6, Cytoscape.js v3.23, ChartJS v4.3, and FileSaver v2.0.5 to visualize the knowledge graphs. The GitHub repository containing the source code of the database is available at https://github.com/mutwil/PlantConnectome_2025version

### API for PlantConnectome

PlantConnectome also has an application programming interface that allows users to conduct search queries remotely. The API accepts GET requests and is implemented using the same set of packages described earlier. For each successful call to PlantConnectome's API, a JSON (JavaScript Object Notation) object is returned, containing the entity-entity relationships and its related information (i.e. definitions, edge basis, original and resolved entity names, etc), pubmed IDs and text summaries associated with the search query. To perform searches using the API, users can add “/api/<search type>/<search query>” to the web address, where “<search type>” and “<search query>” are placeholders representing the type of search and user's query, respectively. <search type>can be normal (keyword/alias), substring (substring), author (author) and title (PubMed ID).

## Supplementary Material

koaf169_Supplementary_Data

## Data Availability

The Plant Connectome database source code is available at: https://github.com/mutwil/PlantConnectome_2025version. The knowledge graph is found at https://plant.connectome.tools/download.

## Supplementary data

The following materials are available in the online version of this article.

**Supplementary Figure S1.** tSNE analysis of the abstracts at different perplexity and iteration values.

**Supplementary Figure S2.** tSNE analysis of the abstracts by different biological processes, as defined by MapMan.

**Supplementary Figure S3.** tSNE analysis of the abstracts by different cellular compartments.

**Supplementary Figure S4.** tSNE analysis of the abstracts by different major organs and cell types.

**Supplementary Figure S5.** Entity resolution pipeline.

**Supplementary Table S1.** Gene IDs (column A) and aliases (column B) used to query the NCBI database for articles.

**Supplementary Table S2.** Journals used to construct the database. The columns indicate the journal name, article title, publication year, PubmedID, and whether the article was analyzed as full text (yes/no).

**Supplementary Table S3.** The three categories, their sub-categories, and keywords used to perform topic analysis of paper abstracts.

**Supplementary Table S4.** Keyword co-occurrence analysis. The two keywords are in columns A and B, and the number of articles in which these keywords co-ocurred are shown in column C.

**Supplementary Table S5.** The prompts used to build the knowledge graph, extract entity definitions and edge basis.

**Supplementary Table S6.** Edge and entity type accuracy evaluation. Each row represents and edge in the knowledge graph. Each edge contains the pubmed ID, source and target nodes, source and target entity types and relationship description. The evaluations of the source types (column G), target types (Column J), and relationship (Column M) are indicated. The comments and the sentences that underpin the edge are found in the other columns.

**Supplementary Table S7.** Evaluation of the edge resolution accuracy. The correctness score is indicated in column E.

**Supplementary Table S8.** Evaluation of the entity type resolution accuracy. The correctness score is indicated in column V.

**Supplementary Table S9.** Evaluation of the triples resolution accuracy. The correctness score is indicated in column K.

**Supplementary Table S10.** Evaluation of the accuracy of the gene regulatory edges (top sub-table) and protein-protein interactions (bottom sub-table).

**Supplementary Table S11.** Evaluation of STRING accuracy. The correctness score is indicated in column E.

**Supplementary Data Set 1.** The code to download and process the papers and generate the figures in the manuscript is available at https://github.com/shanchun001/PlantConnectome_2025version/blob/main/connectome_files/previews/Connectome2025_Figures_June.ipynb.
