# Supplementary material for: PlantConnectome: A knowledge graph database encompassing >71,000 plant articles
Source: Plant Cell. 2025 Jul 23;37(7):koaf169. doi: 10.1093/plcell/koaf169 (PMC12290883; doi:10.1093/plcell/koaf169)
Supplement: koaf169_Supplementary_Data [file koaf169_supplementary_data.zip › Supplemental Figure S1-5.pdf]

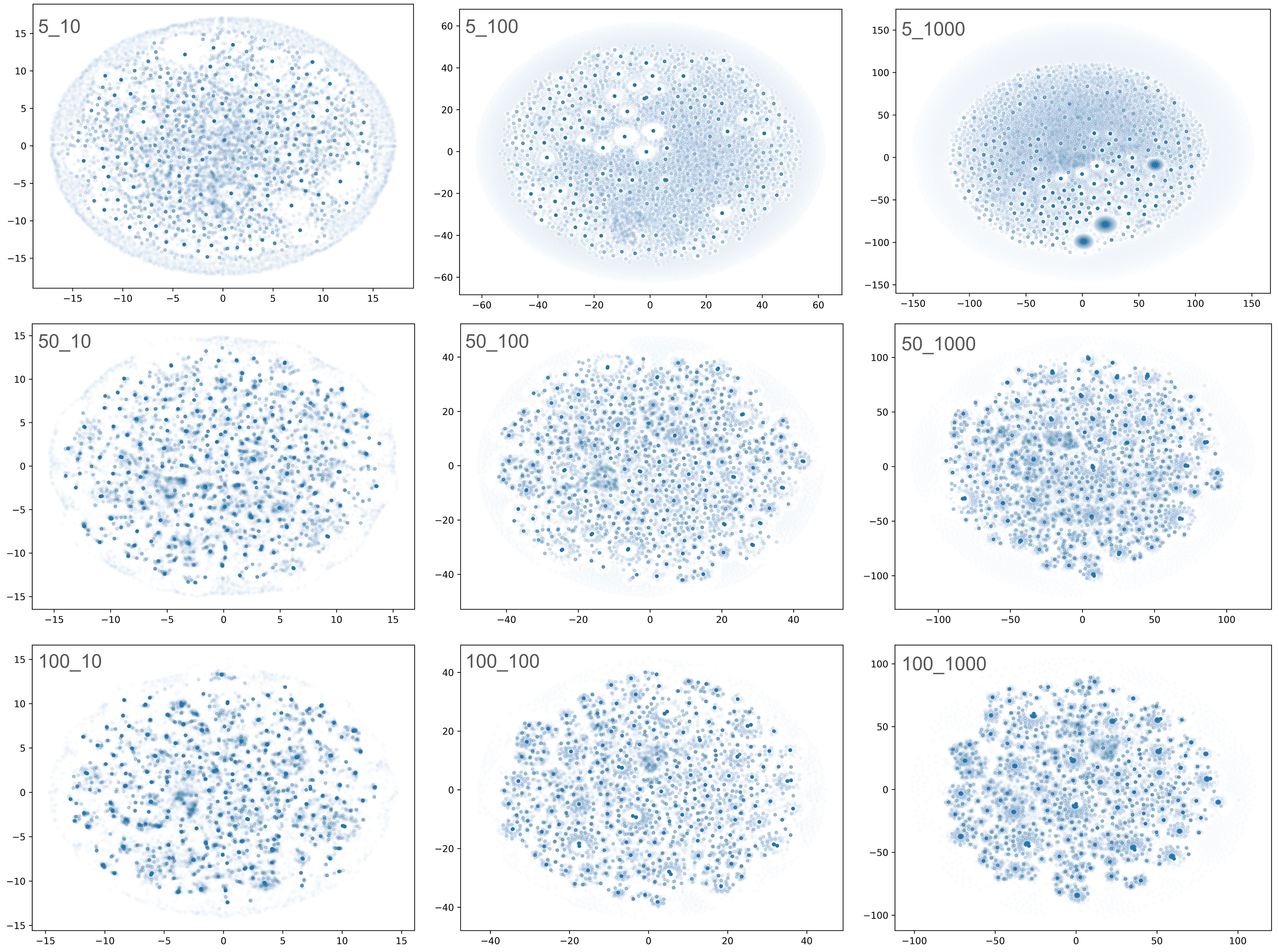

Figure S1. tSNE analysis of the abstracts at the different perplexity and iteration values. The evolution of the plot at a perplexity of 5 (first row), 50 (second row) and 100 (third row) and different ranges of iterations: 10 (first column), 100 (second column) and 1000 (third column). Note that the x- and y-axis values are arbitrary and not labeled, as tSNE embeddings are non-linear projections without interpretable axes.

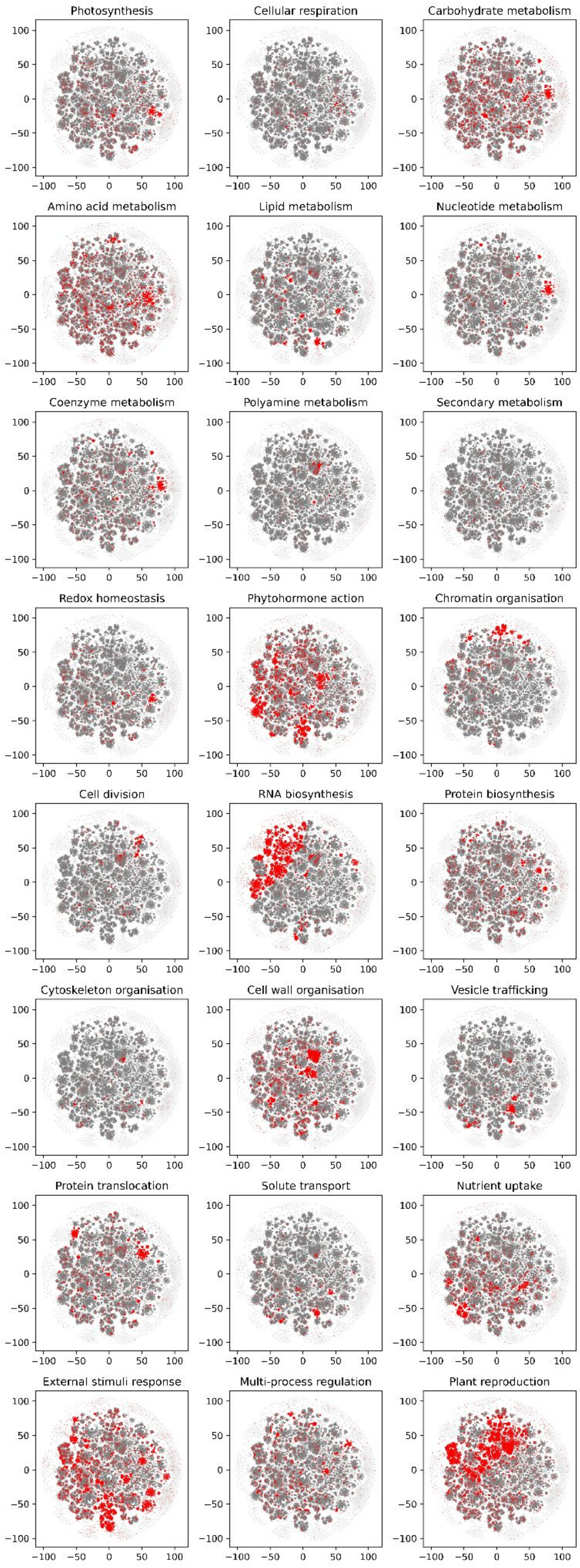

Figure S2. tSNE analysis of the abstracts by different biological processes, as defined by MapMan. A red point indicates an abstract that contains a keyword (e.g., pollen is a keyword for plant reproduction), while a grey point indicates an absence of the keyword match. Note that the x- and y-axis values are arbitrary and not labeled, as t-distributed stochastic neighbor embedding (t-SNE) embeddings are non-linear projections without interpretable axes.

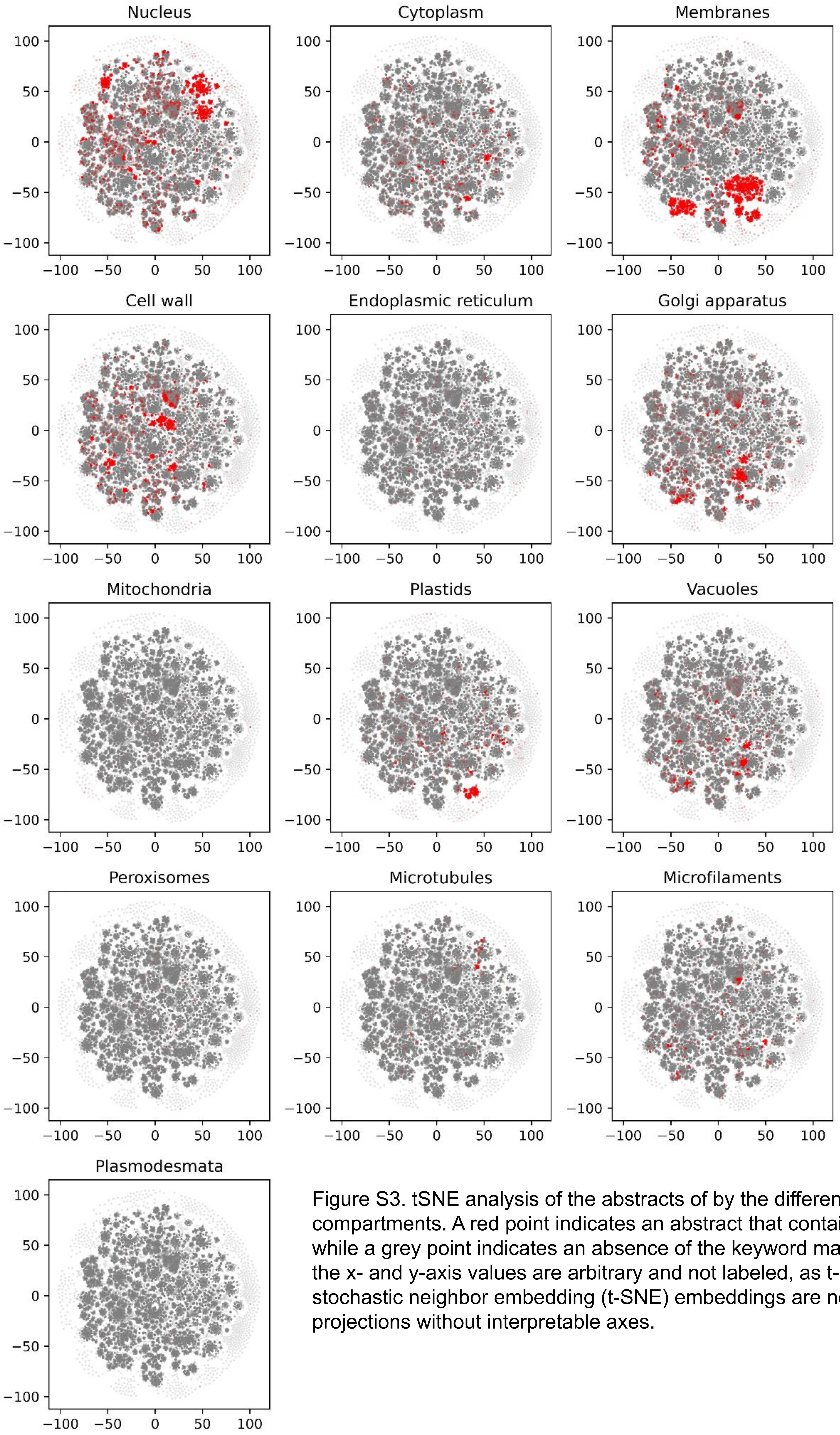

Figure S3. tSNE analysis of the abstracts of by the different cellular compartments. A red point indicates an abstract that contains a keyword, while a grey point indicates an absence of the keyword match. Note that the x- and y-axis values are arbitrary and not labeled, as t-distributed stochastic neighbor embedding (t-SNE) embeddings are non-linear projections without interpretable axes.

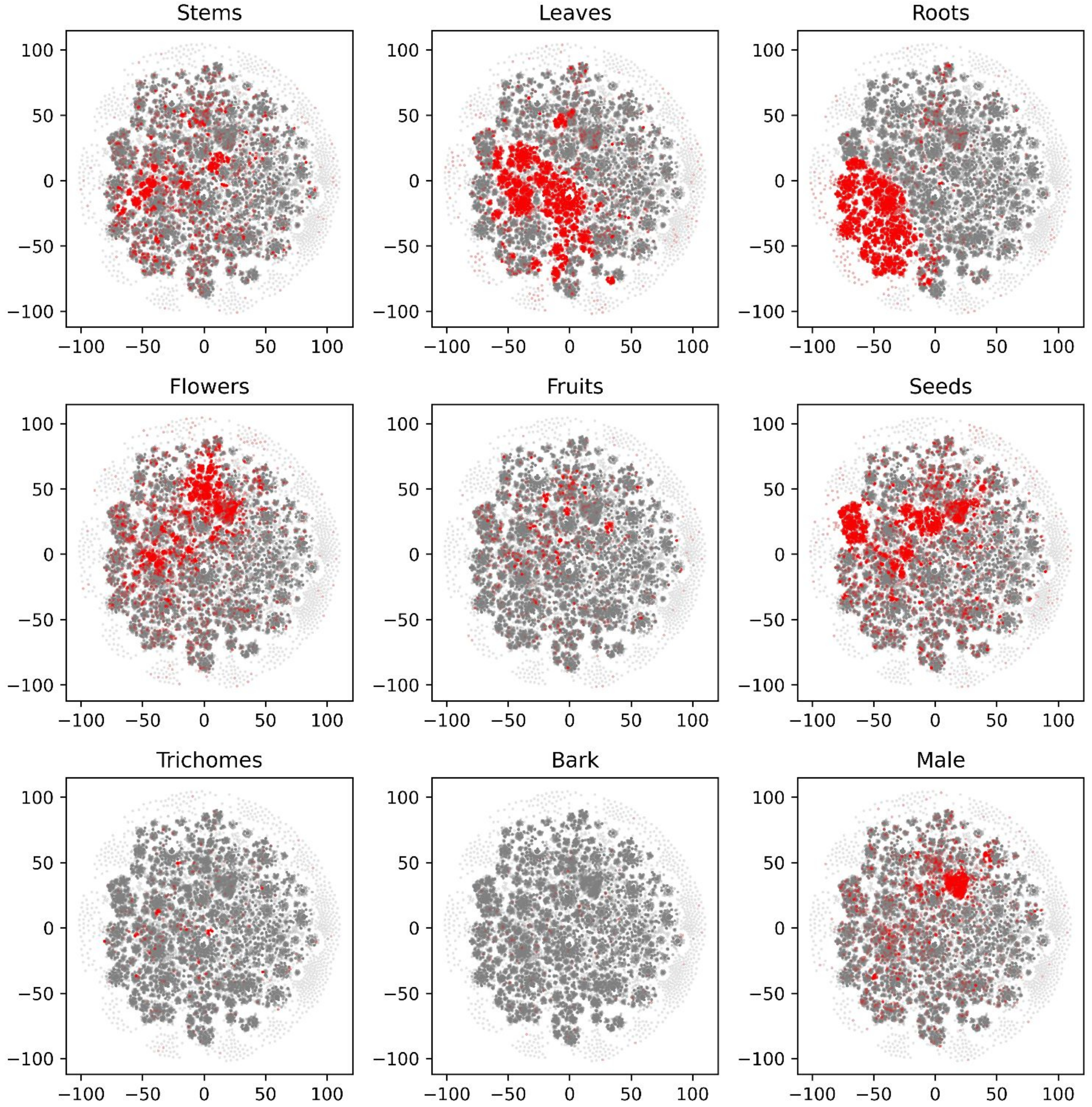

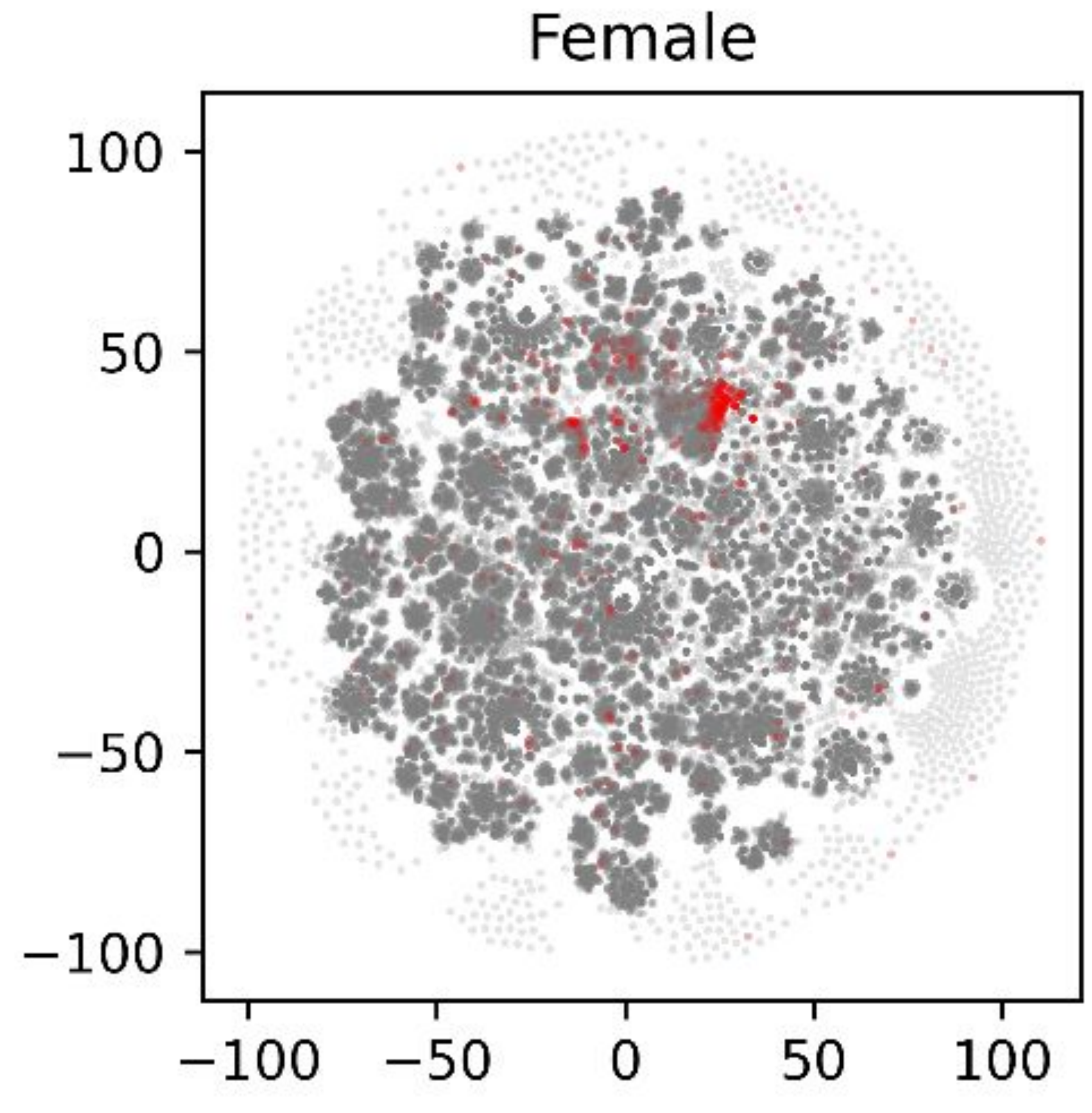

Figure S4. tSNE analysis of the abstracts by different major organs and cell types. A red point indicates an abstract that contains a keyword, while a grey point indicates an absence of the keyword match. Note that the x- and y-axis values are arbitrary and not labeled, as t-distributed stochastic neighbor embedding (t-SNE) embeddings are non-linear projections without interpretable axes.

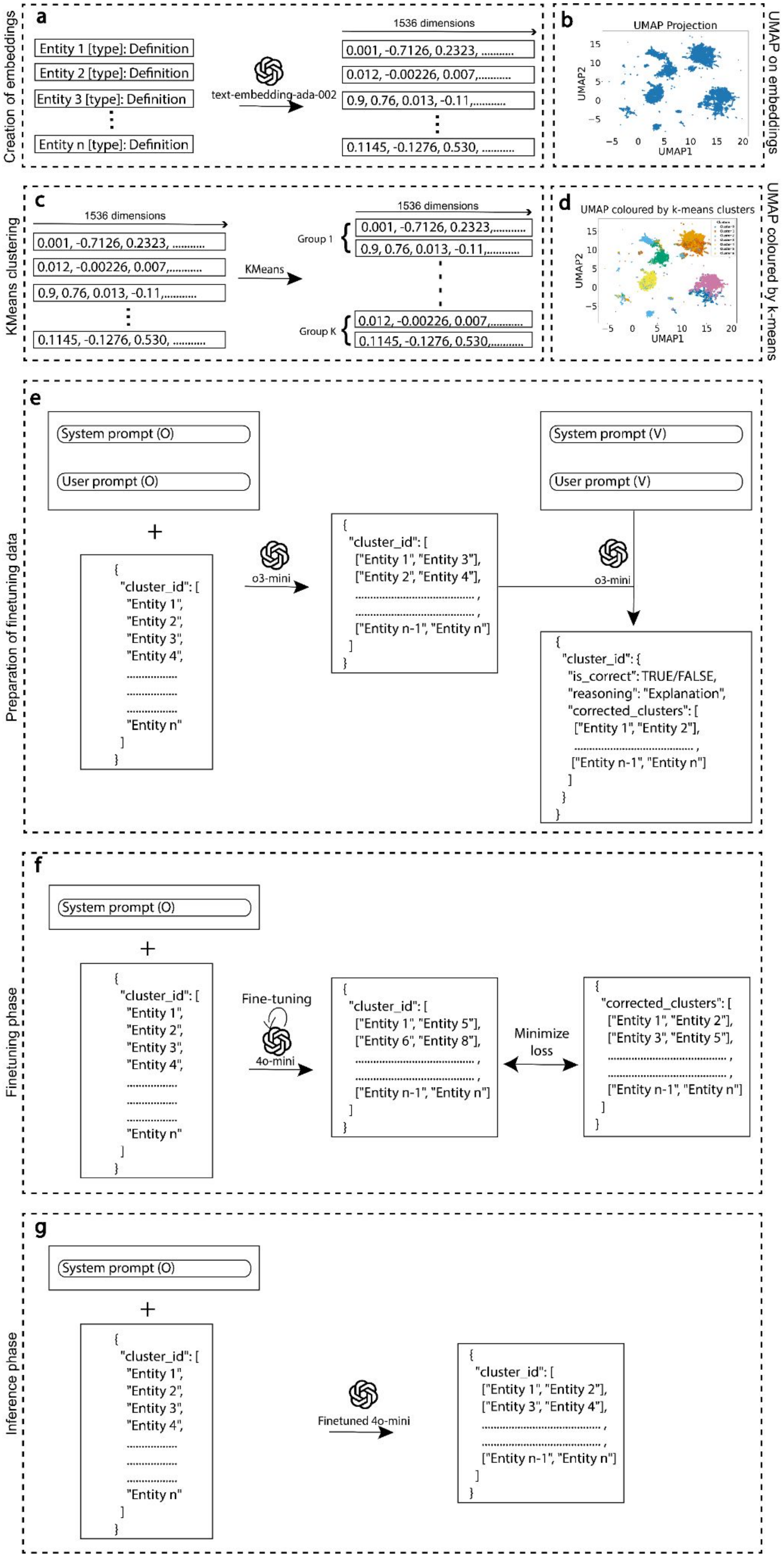

Figure S5. Entity resolution pipeline. a) Creation of embeddings: generating 1,536-dimensional vector representations of each entity - incorporating its type and definition - using OpenAI's text-embedding-ada-002 model; b) Uniform Manifold Approximation and Projection (UMAP) on embeddings: A two-dimensional projection of vector embeddings on a representative subset of the data. Note that the x-axis and y-axis values are arbitrary and represent abstract embedding coordinates; therefore, the axes are unlabeled. c) KMeans clustering: unsupervised grouping of the entity embeddings into initial clusters; d) UMAP colored by KMeans: two-dimensional projection of vector embeddings on a representative subset, colored according to the initial cluster assignments by KMeans; e) Preparation of fine-tuning data: creating and iteratively refining training examples with OpenAI’s o3-mini reasoning model to incorporate corrective feedback; f) Finetuning phase: training openAI's 4o-mini model on the refined dataset to achieve performance comparable to o3-mini; and g) Inference phase: apply finetuned 4o-mini model to the KMeans clusters for finegrained sub-clustering.
